# The Need for a National Accreditation Programme for Professionals Working in the Field of Animal Training and Behavioural Modification in New Zealand

**DOI:** 10.3390/ani10071111

**Published:** 2020-06-29

**Authors:** Lindsay J. Skyner, Kristie E. Cameron, Arnja Dale, Jessica K. Walker

**Affiliations:** 1 Companion Animals New Zealand, Wellington 6141, New Zealand; arnja.dale@spca.nz; 2 Faculty of Health, Education and Environment, Toi Ohomai Institute of Technology, Tauranga 3143, New Zealand; 3 School of Environmental and Animal Sciences, Unitec Institute of Technology, Auckland 1142, New Zealand; kcameron@unitec.ac.nz; 4 Department of Animal Welfare Science and Education, SPCA New Zealand, Auckland 0600, New Zealand; jessica.walker@spca.nz

**Keywords:** behaviour, aversion, reward-based, welfare, standards

## Abstract

**Simple Summary:**

Companion animals play an important role in the lives of New Zealanders. Animals’ guardians often engage professional behaviour and training support to ensure their companion animal’s behaviour is compatible with their lifestyle and expectations. The methods these professionals use vary substantially and potentially subject animals to psychological (and physical) harm where outdated, incorrect or aversive methods of training or equipment are used. The national regulation of training methods or techniques would safeguard the welfare of the animal benefactors of these services. In the absence of such regulation, the development of a national accreditation body may act to protect animal welfare by certifying the experience, qualifications and training methods of the professionals offering these services. We investigated industry opinion and readiness for the establishment of an accreditation body via an online survey and found that accreditation, promoting the use of reward-based training and behavioural modification techniques, was generally supported. We conclude that the establishment of a national accreditation body would ensure that those seeking services source professionals who use best practice when it comes to training and behavioural modification, resulting in lasting benefits to animal welfare.

**Abstract:**

Companion animals are at risk of psychological (and physical) harm if outdated, incorrect or aversive methods of training or equipment are used during training and behavioural modification. Companion animal guardians often engage professional animal behaviour and training services, yet this industry is not regulated in New Zealand. A voluntary national accreditation and registration programme could act to protect the welfare of animals by robustly evaluating the experience, qualifications and training methods of industry professionals. To investigate industry readiness for a national accreditation programme, we conducted an online survey and analysed the responses of 262 animal trainers, behavioural consultants, dog safety educators, veterinarians and veterinary nurses. A national accreditation programme, promoting the use of reward-based training and behavioural modification techniques, was generally supported, especially by individuals holding qualifications and membership of professional organisations. The implementation of such a programme would ensure that those seeking these services are able to source professionals that use best practice when it comes to training and behavioural modification, with lasting benefits to animal welfare.

## 1. Introduction

Animals play an important role in the lives of New Zealanders, with 64.4% of households being home to at least one companion animal [1]. A variety of species are kept as human companions and are expected to behave “well” to fit with societal norms [2], especially where they are invited to live inside the home. This results in a need to train some animal companions (primarily dogs) to perform human-perceived acceptable behaviours and dissuade undesirable behaviours [3], requiring the knowledge of training methods by their guardians. 

Various methods are used to train companion animals, however, some techniques and tools used can be argued as outdated and aversive [4,5]. Disagreement on what constitutes humane methods is ever apparent in the literature [6] and this is mirrored in professional practice. Methods that are considered aversive generally refer to the use of positive punishment and negative reinforcement, as opposed to reward-based training methods of positive reinforcement [7] and negative punishment [3,8]. Negative punishment is sometimes alternatively categorized as aversive [6], this is because for it to be effective, the experience (removal of something the animal wants) must be sufficiently unpleasant to motivate the change in behaviour. 

Companion animals have been reported to be at risk of psychological (and physical) harm with the use of aversive methods of training or equipment [4,6,8,9,10]. The use of shock collars, for example, has been reported to cause burns in dogs [11] along with fear and pain [12], which can have lasting negative associations for the dog’s interactions with humans [12], other animals [13] and the environment [14]. There is little evidence that aversive training is more or as effective as reward-based training [8], yet its usage is still common. A survey of 192 United Kingdom dog owners found 72% used some form of positive punishment [4]. This may be due to an outdated belief that undesirable behaviours (e.g., aggression) in dogs derive from social dominance theory and wolf pack behaviour [3,15]. 

Despite becoming one of the first countries in the world to legally recognize animals as “sentient” [16], there is still a great deal of improvement required to enhance animal welfare standards (and the required positive human and animal relationship), particularly for animals undergoing training [17]. While many professions (e.g. veterinary medicine) are regulated, that is, have a series of rules that must be followed in order to practice, animal training and behaviour modification is currently unregulated in New Zealand, as is the case in the majority of countries around the world [6,10]. This lack of regulation makes it unclear as to the exact numbers of people working in each related profession [10], although anecdotal evidence suggests numbers are on the rise [18].

Accreditation programmes are useful for industries that lack regulation, as they are run by independent bodies and they set standards and certify individuals according to these standards [10]. There is currently no national independent accreditation body to verify the experience, qualification and ethics of individuals working in the animal training industry in New Zealand. The United Kingdom has led by example in this area, with the development of the “Animal Behaviour and Training Council” (ABTC), an accreditation body that promotes humane training and behavioural therapy and lobbying for regulation within this industry (Animal Behaviour and Training Council, http://www.abtcouncil.org.uk/).

Behavioural advice can be sought from a variety of animal professionals. In the United Kingdom, 18% of 192 dog owners surveyed reported that they had obtained help for undesirable behaviours. Of these, 42% had visited a dog trainer, 32% a veterinarian, 26% an animal behaviourist, 9% a veterinary nurse and 9% a friend or relative and a variety of methods were used [4]. The variety of methods and lack of consistency among behaviour professionals in appropriate and correct training techniques indicates a need for public guidance in sourcing appropriate professional help, especially since many professionals available for advice do not have qualifications in the science of learning, the ability to evaluate behaviour or the experience to manage clients and positively impact animals in their care [10]. In the absence of the regulation or knowledge of appropriate professionals for referrals, it is possible that a “have a go” attitude may be encouraged, which could be detrimental and dangerous for all parties. Even veterinarians, who play an important and vital role in educating the public on animal health and welfare, may not have formal courses in animal behaviour [6]. This is particularly concerning, as 72.3% of people considered veterinarians as the best source of information for animal-related issues in New Zealand [1]. 

Although there is no national accreditation body for the animal behaviour and training industry in New Zealand, there are a small number of organisations who offer membership and oversee individuals working in this industry. This is a positive step towards accountability; however, membership is optional and there are inconsistencies in the terminology within ethics and position statements that do not necessarily promote the use of reward-based animal training. At present, there are only two certification schemes available within New Zealand: The Trainer Endorsement Programme, available through the Association of Pet Dog Trainers New Zealand (APDTNZ), and the Veterinary Behaviour Chapter of the Australian and New Zealand College of Veterinary Scientists (ANZCVS). Individuals working in other professions, for example, as behaviour consultants, must seek membership and certification through international groups such as the International Association of Animal Behavior Consultants (IAABC) or Pet Professional Guild (PPG). It appears to be only a matter of time until more certification programmes are developed in New Zealand, especially with the recent publication on the need for global accreditation programmes by Tudge et al. [10] and the reoccurring topic of discussion on training podcasts, social media and other online forums. 

A national accreditation programme should provide a system to protect the psychological welfare of animals undergoing training and behaviour modification and maintain the physical safety of both animals and people [10], such an accreditation programme would offer companion animal guardians a platform whereby they can carefully consider the experience, qualifications and training methods engaged (including any tools or equipment) by industry professionals prior to contracting their services. This will allow both companion animal guardians, other industry professionals seeking to refer clients (e.g. veterinarians, shelter professionals, groomers) and the general public satisfaction that any training technique utilised by accredited individuals holds the long-term welfare of their animal at the forefront and is consistent with the principles of kindness and fairness and the promotion of the fundamental and essential human–animal bond. Once successfully developed and adopted by both industry and the general public, an accreditation programme would have the potential to become nationally regulated, providing a legal requirement to be registered prior to practice for people working in the industry. 

The first step towards developing a national accreditation programme in New Zealand is to establish whether the industry perceives value in such a programme and understand the current methods utilised, and roles performed, by industry personnel. Industry readiness is vital for the success of such a programme, as professionals, along with those seeking services for their companion animals, will need to drive the behaviour change required for accreditation to become common practice. This research investigated industry opinion and readiness through an online survey targeted at trainers, behavioural consultants, dog safety educators, veterinarians and veterinary nurses.

## 2. Materials and Methods

Animal trainers, veterinarians and veterinary nurses were invited to complete a survey during September 2019 via email, social media forums and stakeholder groups (e.g., Dogs New Zealand (Dogs NZ), Association of Pet Dog Trainers New Zealand (APDTNZ), New Zealand Veterinary Nursing Association (NZVNA), New Zealand Veterinary Association (NZVA), Australian and New Zealand College of Veterinary Scientists (ANZCVS)). As there is no membership organisation for animal behaviour consultants or dog safety educators in New Zealand, these professionals were individually sought and sent a survey link. Five web-based surveys, tailored to each of the five professions, were developed in Google Forms (https://www.google.com/forms/about/).

Respondents self-selected their professional role (“animal trainers”, “behavioural consultants”, “dog safety educators”, “veterinarians” or “veterinary nurses”) and completed the appropriate survey. If a respondent identified with more than one professional role, they were able to complete multiple surveys.

Each survey consisted of a maximum of 18 response-dependent questions using multiple choice and open-ended formats. Respondents were asked for their age, highest qualification, gender, location, if they referred clients to other professionals, their membership to relevant clubs, professional bodies and organisations and if they perceived value in accreditation and would be interested in obtaining personal accreditation for their services (see Supplementary Material for a list of common questions).

Not all surveys included all questions, as some were not appropriate for each targeted profession. For instance, additional questions were asked of the animal trainers to assess their role in animal training, e.g., whether they provided one-to-one or group training of animals for clients. 

In addition, there was an opportunity for respondents to comment on their behavioural qualifications, the qualities they perceive as necessary for accredited professionals and general ideas about accreditation, however, the latter was not analysed due to the extreme variety of comments. 

A descriptive quantitative analysis of the survey data was conducted using SPSS^®^ (Version 22, IBM Corp.) with additional chi-square analyses used to measure associations between variables. A basic thematic analysis based upon that of Saldana [19] was conducted by two authors on the comments given by respondents regarding behavioural qualifications and the qualities required for working in the animal behaviour industry. Human ethics approval was obtained from Unitec Research Ethics Committee (#2019-1026).

## 3. Results

A total of 483 self-identified professionals responded to the online survey request and answered the survey tailored to their profession. In order to streamline the data analysis, and to measure the responses of those professionals that are possibly eligible for accreditation in the future, only responses from individuals currently offering a training or behavioural service were analysed. This resulted in analysing 173/197 surveys from animal trainers, 15/15 behavioural consultants, 18/18 dog safety educators, 20/68 veterinarians and 36/185 veterinary nurses. For those veterinarians and veterinary nurses not currently offering a behavioural service, there was considerable value perceived in an accreditation process, with 91.7% (44/48) of veterinarians and 91.2% (135/148) of veterinary nurses that offered an opinion showing support for recognising professionals with accredited skills.

### 3.1. Interest in Accreditation

Overall, 63.0% (165/262) of respondents reported an interest in accreditation, 9.5% (25/262) responded that they were not interested and 26.0% (68/262) were unsure (five respondents did not answer). Of the interested respondents, 98 were animal trainers, 10 were behavioural consultants, 16 were dog safety educators, 9 were veterinarians and 32 were veterinary nurses. A chi-square test of independence revealed a significant association between professional role and interest in accreditation (*χ*^2^ (8, *N* = 256) = 22.68, *p* < 0.001). The Cramer’s V (a measure of effect size of the association between the two variables with more than two levels, see (Cohen, 1988)) was moderate (*V* = 0.30). Animal trainers were the largest group who reported they were uninterested or unsure about accreditation (43.0% (74/173), whereas across all other professions, less than 13.4% (19/89) were not interested or unsure about accreditation.

### 3.2. Behavioural Modification Methods

**Figure 1:**
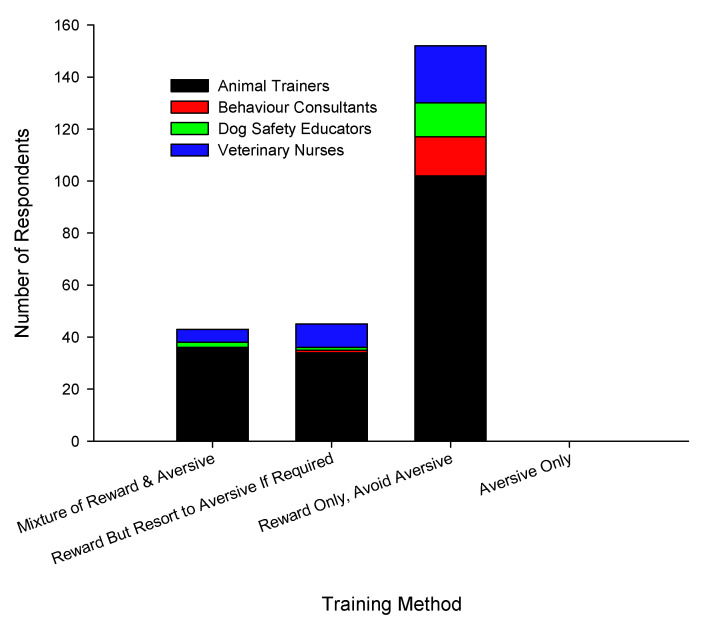
Number of respondents that reported using different training methods: A mixture of reward and aversive methods, reward but would resort to aversive methods if required, reward only and avoided aversive methods and aversive only.

The majority of respondents reported using only reward-based training and avoided using aversive methods (Figure 1), that is, 59.3% (102/172, one did not answer) of animal trainers, 93.8% (14/15) of behavioural consultants, 81.3% (13/18, two did not answer) of dog safety educators and 61.1% (22/36) of veterinary nurses. Veterinarians were not asked this question. Animal trainers reported using a mixture of reward-based and aversive training (20.9%, 36/172) and used reward-based training as much as possible, only resorting to aversion if all else failed (19.8%, 34/172). A few veterinary nurses reported using a mixture of methods (13.9%, 5/36) and reward-based with aversive methods if necessary (25.0%, 9/36). No respondents reported that they solely used aversive methods.

### 3.3. Animals

Of the 173 animal trainers, 29.5% (51/173) reported working with a particular species; 88.2% (45/51) with dogs, 7.8% (4/51) with horses and 3.9% (2/51) with cats. Of the 36 veterinary nurses, 77.8% (28/36) reported working with one particular species; 89.3% (25/28) with dogs, 42.9% (12/28) with horses, 17.9% (5/28) with rabbits and rodents, 10.7% (3/28) with cats and 10.7% (3/28) with birds or exotics.

### 3.4. Roles

Animal trainers were asked about their method of teaching clients to train their animals. Of the 173 trainers, 74.0% of trainers taught in a group class setting and 54.3% of trainers taught clients on a one-to-one basis; 36.8% of trainers performed both teaching methods. Nearly one third (30.6%) of trainers reported using behavioural modification techniques on animals on a one-to-one basis and 37.0% of trainers reported teaching their clients behavioural modification techniques. Training animals for clients was carried out by 28.9% of trainers and 30.1% of trainers reported working with clients on specialised problem behaviours. Only 13.9% of animal trainers reported performing all types of training of animals and teaching of clients. There was a significant association between interest in accreditation and the role of dog trainer (*χ*^2^ (10, *N* = 439) = 69.38, *p* < 0.001, *V* = 0.40); between 63.8% and 78.8% of trainers who taught across all types were interested in accreditation, with a large proportion of trainers who solely teach group classes being interested (57.0%), unsure (10.2%) or not interested (32.8%) in accreditation.

Veterinary nurses reported performing behavioural training services in their role: Of the 36 nurses, 72.2% provided puppy preschool, 22.2% provided behavioural consultations, 19.4% reported providing animal training and 5.5% reported treating problem behaviours. Nearly half (45.0%) of the 20 veterinarians provided details on the type of behavioural services they offer clients, with 30% providing behavioural consultations, 15.0% providing animal training and 5.0% assessing or treating problem behaviours. Nearly all (93.3%) of the 15 animal behavioural consultants responded, with 80.0% providing training and behavioural consultations for animals such as dogs, cats or horses and 33.3% treating behaviour issues such as anxiety or aggression. 

### 3.5. Demographic Information

**Figure 2:**
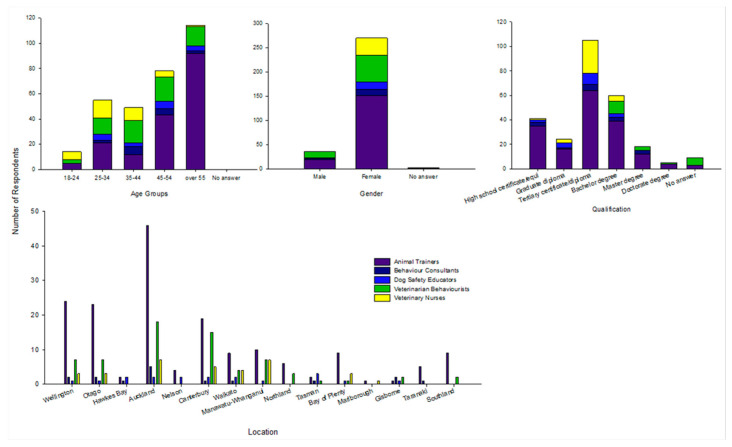
Demographic data across all professional groups (animal trainers, behaviour consultants, dog safety educators, veterinarians and veterinary nurses) describing age, gender, qualification and location.

The age, gender, qualification and location of the professionals are graphed in Figure 2. The majority of the 260 respondents (that answered) were female (88.8%, 231/260). Only 10.3% (27/260) of males answered and 19 of these respondents were identified as animal trainers. Most respondents were over 55 years old (41.1%, 108/262) or between 45–54 years old (24.3%, 64/262). 

The greatest percentage of individuals working in the industry of training and behavioural modification across all professions held a tertiary certificate or diploma (40.1%, 105/262) or bachelor’s degree (22.9%, 60/262), with 15.7% (41/262) of respondents holding a high school certificate or equivalent; 35 of these respondents were working as animal trainers. There was a significant association of qualification and interest in accreditation (*χ*^2^ (2, *N* = 248) = 11.14, *p* = 0.004, φ = 0.21) of those with high school certificate or equivalent compared to those with higher qualifications.

Across professions, 257 respondents elected to include their qualification, with 247 respondents also giving an indication of interest in accreditation. Those holding qualifications of a tertiary certificate level or above (185 respondents across professions) reported more interest in accreditation (68.1%, 126/185), 6.5% (12/185) were not interested and 25.4% (47/185) were unsure. Whereas for those with a high school certificate, 64.5% (40/62) reported an interest in accreditation, 14.5% (9/62) were not interested and 21.0% (13/62) were unsure.

### 3.6. Referring a Client

A large percentage of animal trainers (76.3%, 132/173) reported that they refer clients to animal behaviourists or veterinarians specialising in behaviour. Of these trainers, 90 reported they would prefer to recommend animal behaviourists or veterinarians holding a national accreditation and 39 trainers reported no preference in recommending a trainer with a professional accreditation. Of the behavioural consultants, 73.3% (11/15) reported that they refer their clients when required with nine of these consultants preferring to refer to future accredited professionals. Of the dog safety educators, 86.7% (15/18) refer clients when required, with 13 preferring to refer to future accredited professionals. Nearly all veterinarians (85.0%, 17/20) and veterinary nurses (97.2%, 35/36) reported that they refer their clients when required, with 16 and 34, respectively, preferring to refer to future accredited professionals. 

There was a significant association between profession and referring clients (*χ*^2^ (4, *N* = 252) = 17.58, *p* < 0.001). This means that individuals are more likely to refer clients to future accredited professionals (79.8%, 201/252, 10 did not answer) rather than refer to unaccredited professionals (20.2%, 51/252).

### 3.7. Membership to an Organisation

**Figure 3:**
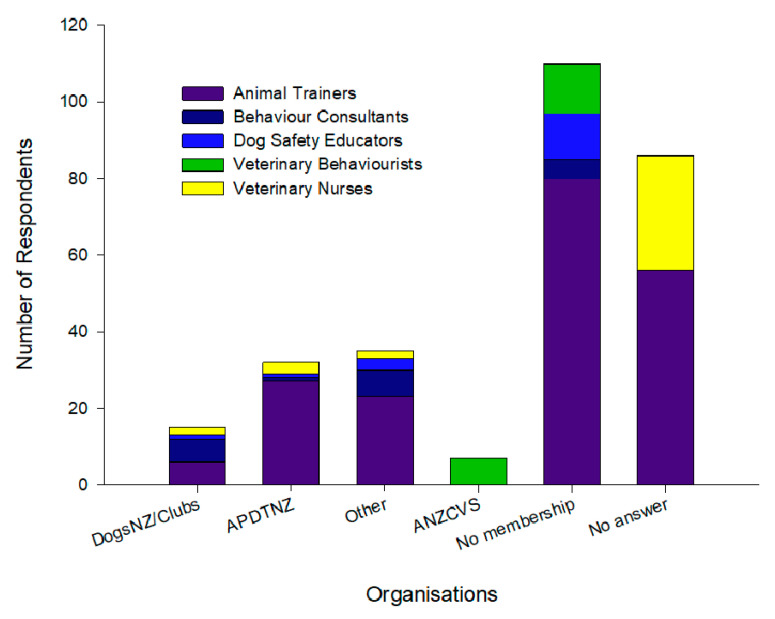
Number of respondents belonging to Dogs New Zealand (DogsNZ)/clubs, Association of Pet Dog Trainers New Zealand (APDTNZ), other or Australian and New Zealand College of Veterinary Scientists (ANZCVS), those with no membership or that did not answer across profession (animal trainers, behaviour consultants, dog safety educators, veterinarians and veterinary nurses).

Half of the respondents offering a behavioural service reported to be members of an animal training club or organisation (9.6%, 130/262), and were made up of 101/173 animal trainers, 10/15 behavioural consultants, 6/18 of dog safety educators, 7/20 veterinarians and 6/36 veterinary nurses (Figure 3). Across all respondents 33.2% (87/262) had memberships and were interested in accreditation, 2.7% (7/262) were not interested and 23.4% (35/262) were unsure (one did not answer). Conversely, 29.8% (78/262) of respondents were not members of an organisation but interested in accreditation, 6.5% (17/262) were not interested and 12.2% (32/262) were unsure (five did not answer; Figure 3). 

The majority of animal trainers with organisational membership supported accreditation (58.4%, 101/173), with 19.9% (34/101) with organisational membership not supporting or unsure about accreditation. Of the trainers that did not hold organisational membership, 31/69 (one did not answer) were interested in accreditation and 38/69 were not or unsure. Most of the animal trainers who reported specific membership to organisations (65/57) belonged to APDTNZ (41.5%, 27/65), but not exclusively. Of these, 77.8% (21/27) reported interest in obtaining accreditation, with 1/27 not interested and 5/27 unsure. In contrast, 33.38% (22/65) were members of a dog club or the umbrella organisation Dogs New Zealand. Of these, 31.8% (7/22) reported interest in obtaining accreditation, while 2/22 were not interested and 13/22 were unsure. The remaining trainers (24.6%, 16/65) were members of other companion animal-related organisations.

### 3.8. Qualitative Analysis

#### 3.8.1. Behavioural Qualifications

The most common qualification reported across animal trainers, dog safety educators and behavioural consultants (14.1%, 21/149 reports) was a paper previously offered by Massey University, New Zealand (“Principles of Canine Behaviour”). Meanwhile, 15.7% (13/89) of animal trainers that provided comment mentioned practical experience as a qualification. Many animal trainers reported undertaking the Certificate of Canine Behaviour and Training at Unitec, the only provider of the New Zealand Qualifications Authority (NZQA) accredited course (9/89), and attending workshops or seminars (8/89). A smaller number of animal trainers completed a Delta Institute certificate (4/89, offered from Australia), the Karen Pryor Academy Certified Training Partner certificate (3/89) or other online courses (3/89). The most common for behaviour consultants, although completed by a small number of respondents, was the Susan Friedman Living and Learning with Animals course (3/24), the Hills^®^ Puppy Preschool (a pet food-sponsored programme) by veterinary nurses (5/13) and ANZCVS Veterinary Behaviour Chapter membership (4/14) by veterinarians. Numerous online certificates were completed by members of each profession.

#### 3.8.2. Required Qualities for Animal Behaviour Professionals

A basic thematic analysis [19] identified that the most common qualities identified as required by the behaviour consultants, dog safety educators, veterinarians and veterinary nurses was training expertise, knowledge and understanding. It was also reported by all the professional groups that animal behaviour professionals should also have relevant qualifications and a personality that includes patience, empathy, kindness, honesty, passion and calmness. Professional currency was also considered important.

## 4. Discussion

This research aimed to investigate industry opinion and readiness for a national animal behaviour and training accreditation programme in New Zealand. The results have shown that promoting the use of reward-based training and behavioural modification techniques is supported by industry professionals in New Zealand. Accreditation allows for consumers to identify an individual offering a behavioural service that has satisfied a series of professional qualities, knowledge and abilities, affording them a national recognition beyond that of belonging to individual animal-related clubs and organisations. In the future, this could be regulated to offer further accountability for training and behavioural modification services provided within the growing companion animal industry in New Zealand. 

The results indicate a similar percentage of respondents were interested in accreditation that hold memberships (33%) as those that did not (30%). An association was identified between an individual’s professional role, their interest in accreditation and their level of education. Sixty-eight percent of individuals with a tertiary qualification were supportive of accreditation compared to nearly 65% whose highest qualification was a secondary school graduate diploma. 

Animal trainers were more likely to report engaging aversive training methods if required. Despite the majority (57%) of this profession showing support for an accreditation programme in general, a higher percentage of trainers, compared to other professional roles, showed a lack of interest in the programme having a reward-based ethical stance. Branson et al. (2009), as cited in [6], found that “working dog” trainers who were educated were more likely to use positive reinforcement methods, and that the use of aversive methods (correction and shock collars) was more likely by trainers with lower levels of education. 

As the proposed accreditation programme is based on the use of reward-based training techniques, accreditation can provide a level of credibility to those lacking a university qualification but who engage in continuing professional development (CPD) or have ample practical experience. The caveat, however, is that individuals must seek to be accredited for their behaviour service and our data suggest those without qualification, who are over 55 years old and use aversive methods are not interested nor do they perceive value in such a scheme. 

Most respondents reported a preference to be able to refer or recommend other professionals that were accredited. Of the animal trainers, however, only 57% reported being personally interested in gaining national accreditation, the majority of whom already regularly referred clients to animal behaviourists and/or veterinary behaviourists. The difference in accreditation interest between professional roles could demonstrate inconsistency in professional ethics and a possible lack of recognition of an individual’s limitations. It is important, therefore, to factor in requirements for referring when developing accreditation standards and codes of ethical conduct to ensure professionals are not acting beyond their experience or qualification level, which could be detrimental to the animals and people involved [10].

Veterinarians are a group that receive minimal training in animal behaviour [6], yet in our survey, veterinarians and veterinary nurses reported carrying out behavioural consultations and facilitating puppy preschool for clients as their main behavioural services. Better education for professionals, such as veterinarians (and other professions like those surveyed), in animal behaviour, would not only improve their ability to recognise behavioural signs of fear, anxiety and stress in dogs (behaviours that are often missed by guardians [4,20]), but also assist in their understanding of when referral to other qualified professionals that use reward-based training techniques is required [7]. Animal professionals working in the training and behavioural modification industry must possess the ability to interpret an animal’s state of wellbeing and welfare, including health and behaviour, in order to assess whether the methods being used are humane [6] and therefore align with the prescribed skills and ethical stance of accreditation. 

Animal trainers were asked about their method of delivering training, for example, whether they taught individually (e.g., one-to-one with a guardian) or in a group (e.g., obedience classes). Interestingly, only 57% of the animal trainers that taught group classes were interested in accreditation, compared on average to 74.1% of those offering other training types (such as specialised training or behavioural modification) as well. The animal trainers offering group instruction could be considered an “at risk” group, where there is considerable contact with companion animal (largely dog) guardians, yet they are unwilling, or not able, to satisfy the requirements of accreditation. This group of professionals, therefore, requires further attention to formulate a plan to attract these practitioners to the value of gaining the ability to be accredited for their clients, both human and animal, to ensure our community has animals with the optimal welfare associated with reward-based training.

The limitations of this research include potential risks to reliability, as respondents were required to self-select their survey. A large number of veterinarians and veterinary nurses responded to the survey but did not offer behavioural services and these data were excluded from the qualitative analysis. The qualitative data of all respondents were analysed to elucidate a generalised “opinion” of those professionals *already* offering behavioural services (therefore having the potential to be eligible for accreditation), as well as those not eligible but who could refer to services offered by accredited professionals. It was difficult to discount such a large sample from the quantitative analysis, however, it should be noted that this population did express that accreditation is valuable and provided scope for the future measurement of behavioural services in these industries and potential CPD to gain accreditation. Another limitation of the analysis was that professionals may have answered in favour of social bias so as not to present themselves in a negative light. Although internet surveys are considered to be less at risk of desirable responding in comparison to other survey methods [21], where respondents reported on their training methods, individuals may have been reluctant to admit to using aversive methods. It is recommended that the future assessment of industry palatability for accreditation programmes includes precise descriptions of the roles of animal trainers and methods used to account for the noted inconsistencies surrounding the definition of reward-based and aversive training techniques. 

In summary, the development of a national accreditation programme for professionals working in the animal training and behavioural modification industry in New Zealand will provide some much-needed clarity for both professionals and companion animal guardians on the experience, qualifications and ethics of individuals. At the same time, accreditation will provide a “badge of honour” for those who are successful in achieving it. Furthermore, aligning relevant tertiary education programmes with accreditation standards will enhance the credibility of scientific and reward-based methods. Professionals and those seeking services can be assured that employing accredited individuals ensures they use best practice in the assessment of applied behaviour when it comes to training and behavioural modification, with lasting benefits to animal welfare. Industry acceptance and understanding is vital to ensure the success of national uptake and it is likely that marketing and education will be of great importance to successful establishment.

## Supplementary Materials

The following are available online at https://www.mdpi.com/2076-2615/10/7/1111/s1.
