# Supplementary material for: The Need for a National Accreditation Programme for Professionals Working in the Field of Animal Training and Behavioural Modification in New Zealand
Source: Animals (Basel). 2020 Jun 29;10(7):1111. doi: 10.3390/ani10071111 (PMC7401583; doi:10.3390/ani10071111)
Supplement: Supplementary file 1 [file animals-10-01111-s001.pdf]

Supplementary Information:

Common Survey Questions

1. Are you an ____ with an interest in behaviour?
2. Do you currently offer specialised services in the field of behaviour?
3. What do these services include?
4. Which species do you work with?
5. Do you:
    a. Only use reward-based training and avoid aversive methods
    b. Use reward-based training as much as possible and only resort to aversives if all else fails
    c. Use a mixture of reward-based and aversive training
    d. Only use aversive training
6. Do you ever refer or recommend ___ to clients?
7. Would you prefer to recommend ___ that have been accredited by a national accreditation body for their use of reward-based methods/qualifications/experience?
8. Are you a current member of any animal behaviour/training organisations either in NZ or abroad? Which ones?
9. Do you think an accreditation for ___ specialising in behaviour would be valuable in NZ?
10. What qualities/skills would you like to see in a ___ that has gained accreditation?
11. Would you be interested in gaining accreditation as a __ from a national accreditation body?
12. Demographics:
    a. Age group
    b. Gender
    c. Location
    d. Highest qualification
13. Please provide details of any qualifications related to behaviour and training that you hold
